# On “Five-Minute Papers”

**Published:** 1897-02

**Authors:** Charles M’Manus


### ON "FIVE-MINUTE PAPERS."[1]

BY CHARLES M'MANUS, D.D.S.

When I was asked to join that forlorn hope, the minute—five-minute—men of dental meetings, I accepted at once, because I believe it to be a duty every member (particularly every young member) owes to any society to which he may have the honor to belong to help along the meetings and fill out the programmes to the best—or the worst—of his ability.

It was suggested that I undoubtedly could not have anything practical to say, but that I might ramble on about things in general and "short papers" in particular, and that I should be doing a service to the other speakers by affording a contrast.

I am rather fond of five-minute papers,—most people are,—because, in the first place, they are comparatively short, and when one begins, the audience and the speaker are able to feel a sense of relief in the thought that it will be all over in five minutes.

If one has some things of practical interest to bring before a meeting, it would seem as if these short papers and talks were just the place to introduce them. And if one has nothing at all to say, it will not take anything like five minutes to demonstrate the fact.

Lest I might be accused of being more presumptuous than I really am, I want to say right here that the little I have to offer is addressed to the very young men,—I wish there were more of them here,—the young men early in practice and just joining their local societies, the men from whose ranks will have to be recruited the dental societies of the twentieth century, and from among whom may be expected to come the reluctant essayists of the future.

It is not such an easy matter to get men to take an active part in a dental meeting. You will observe on your programme that not a single one of the four papers is written by a man on the membership roll of the Association. At a meeting of another society, out of one hundred members it was impossible to secure more than two men willing to read papers.

That this state of affairs is not confined to any particular locality will not be questioned by any one who has given the subject attention.

---

[1] Read, with thirteen others, at the meeting of the Northeastern Dental Association, Springfield, Mass., Thursday, October 22, 1896.

I have no sympathy for the young man who says he cannot contribute a short paper, or talk to his local society meeting, because he has nothing of real interest to say. If we only opened our mouths when we had something of great value to put forth, what a very quiet world this would be—even during a Presidential campaign.

While a very modest young dentist might think it presuming to burst out with a real live dental essay about something he hopes might interest his fellow-practitioners, he ought to have no hesitation about joining the "five-minute men."

How often do we hear of some point in practice or method of procedure brought up at a meeting or published in a journal, and immediately men appear from all sides to say that they did the very same thing in 1870 or 1860 or 1850, as the case may be, but that they did not happen to think to say much about it.

The place to bring forward these little points and "get a record" is in the "five-minute" paper.

One should never hesitate about reading a short paper because he is afraid it might be criticised harshly; he is fortunate if it be discussed at all.

To have a paper really discussed, criticised, pulled to pieces, and fought over would be a great compliment to any young man. The usual fate of the ordinary dental essay is to have it fall to the floor with a dull, cold thud.

A well-known writer in the course of a recent article says,—

"The usefulness of the older-fashioned State societies is passing away, and this decline is due to the decline of the old-fashioned essay and clinic. The rambling essay of the amateur writer of former days is giving way to the highly wrought monograph of the specialist."

I think that if one had time to go over the old files of the dental magazines closely he would find that "the amateur writer of former days" and the "highly-wrought specialist" of to-day are, in many cases, one and the same person.

It is simply a case of evolution, for, like Topsy, they have "growed." Many of the more gifted writers in dental literature to-day began with very modest efforts, and I am sure that that is likely to remain a condition until men are born dental specialists and not infants.

As a bit of mental discipline it would be an excellent idea for the young dentists to form a habit of writing up (in their moments of enforced leisure) any cases of interest that may occur in their practice, not simply with the chastened hieroglyphics of the case-book, but with an eye towards a slight literary style and finish and scientific edge strength. Not necessarily for publication either, but as an evidence of good faith and interest in their profession outside the bread-and-butter aspect.

In fact, I should think that some such beginning would be a sort of literary "school of technics" for young dentists that might in the future blossom forth and bear fruit on the announcement programmes of their societies.

And so my earnest advice to the young dentist is, join the patriotic ranks of the "five-minute men." Begin there, and the day may come when you too can produce "the highly-wrought monograph of the specialist," which often, like "Mercutio's soul, is but a *little way* above our heads."

